# Supplementary figures and images for: Comparison of alemtuzumab, anti-thymocyte globulin, and post-transplant cyclophosphamide for graft-versus-host disease and graft-versus-leukemia in murine models
Source: PLoS One. 2021 Jan 11;16(1):e0245232. doi: 10.1371/journal.pone.0245232 (PMC7799789; doi:10.1371/journal.pone.0245232)

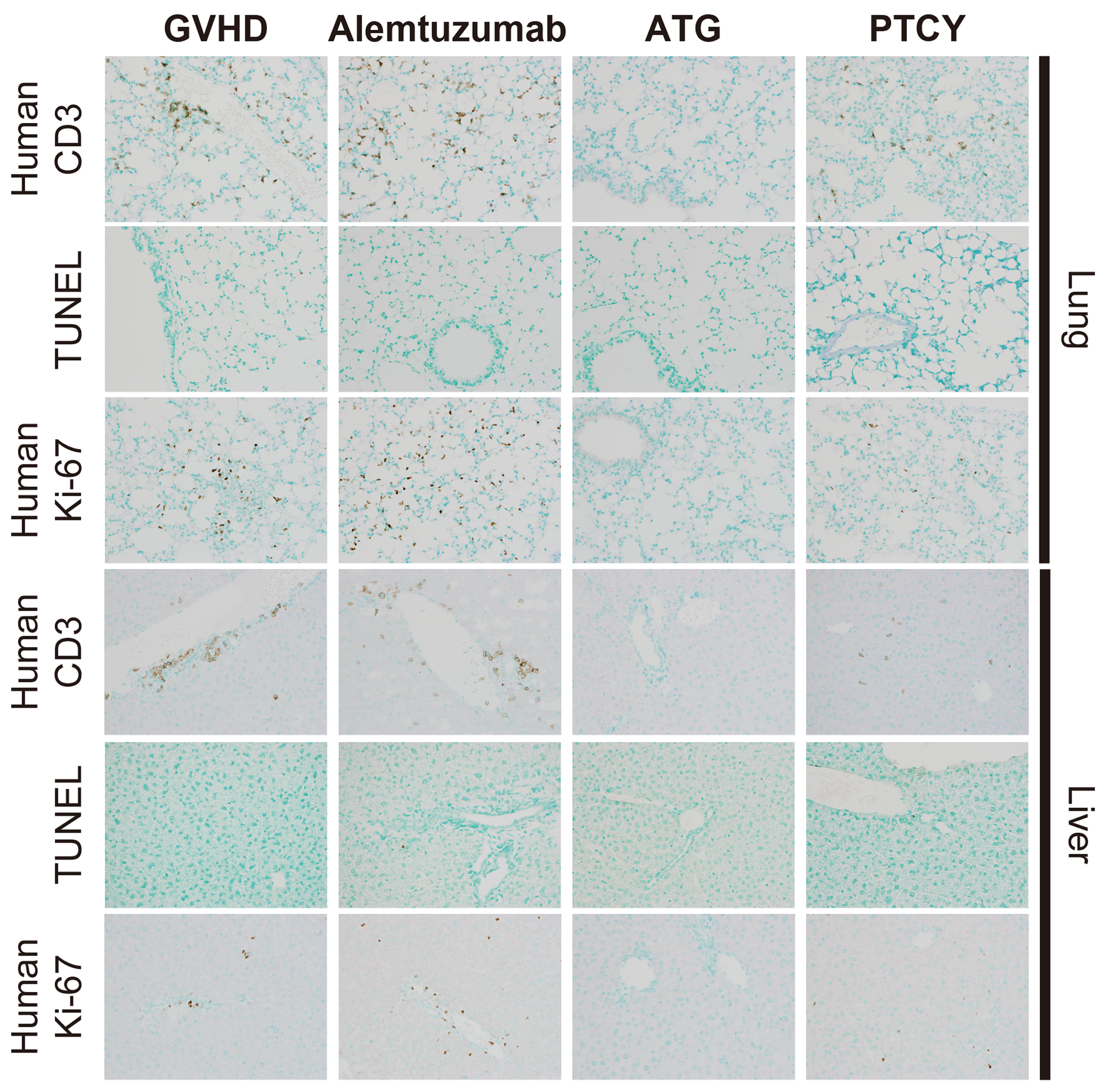

Supplement: S1 Fig — Sections of lungs and liver in the mice were stained with human CD3, Ki-67, and TUNEL and detected with DAB. Methyl green was used for a nuclear counterstain. (TIF) [file pone.0245232.s001.tif]

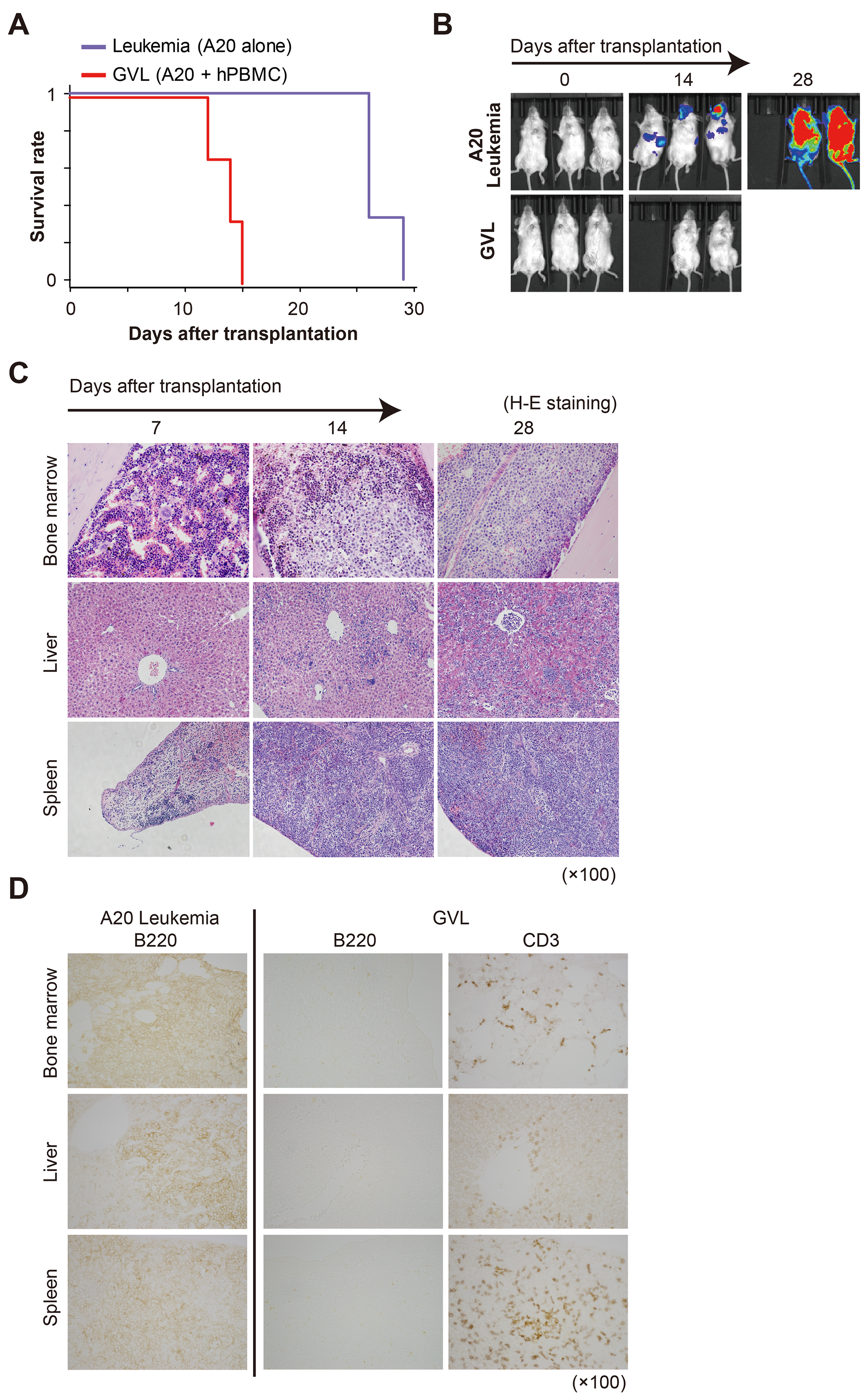

Supplement: S2 Fig — 2×10^3^ luciferase-transfected (Luc) A20 cells were transplanted into the irradiated mice to create the leukemia model, and 2×10^3^ Luc-A20 cells with 5×10^6^ hPBMCs were co-transplanted to create the GVL model. Each group consisted of three mice. They were observed daily to assess survival (A), and tumor growth was detected using bioluminescence imaging (B). The histopathological analysis also showed A20 tumor growth. Sections of the bone marrow, liver, and spleen of the leukemia mouse model were stained with hematoxylin and eosin (C). Immunohistochemistry staining with anti-B220 (mouse B cell) and anti-human CD3 detected A20 tumor and hPBMC invasion, respectively (bone marrow, liver, and spleen) (D). (TIF) [file pone.0245232.s002.tif]

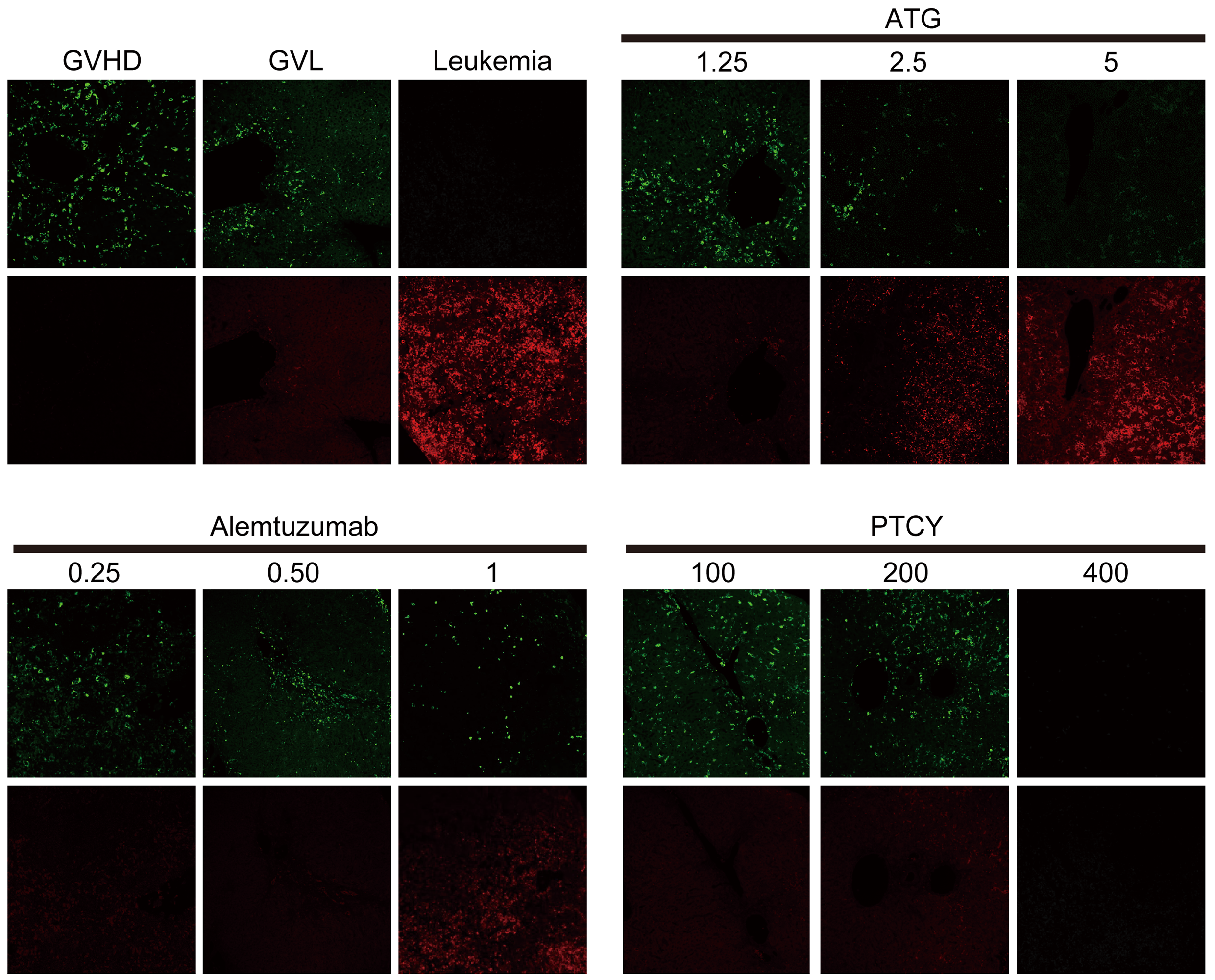

Supplement: S3 Fig — Sections of the lungs and liver in mice were stained with B220 (Fluorescein, green) and human CD3 (Cyanine 3, red) and detected by fluorescent immunohistochemistry with tyramide signal amplification. (TIF) [file pone.0245232.s003.tif]

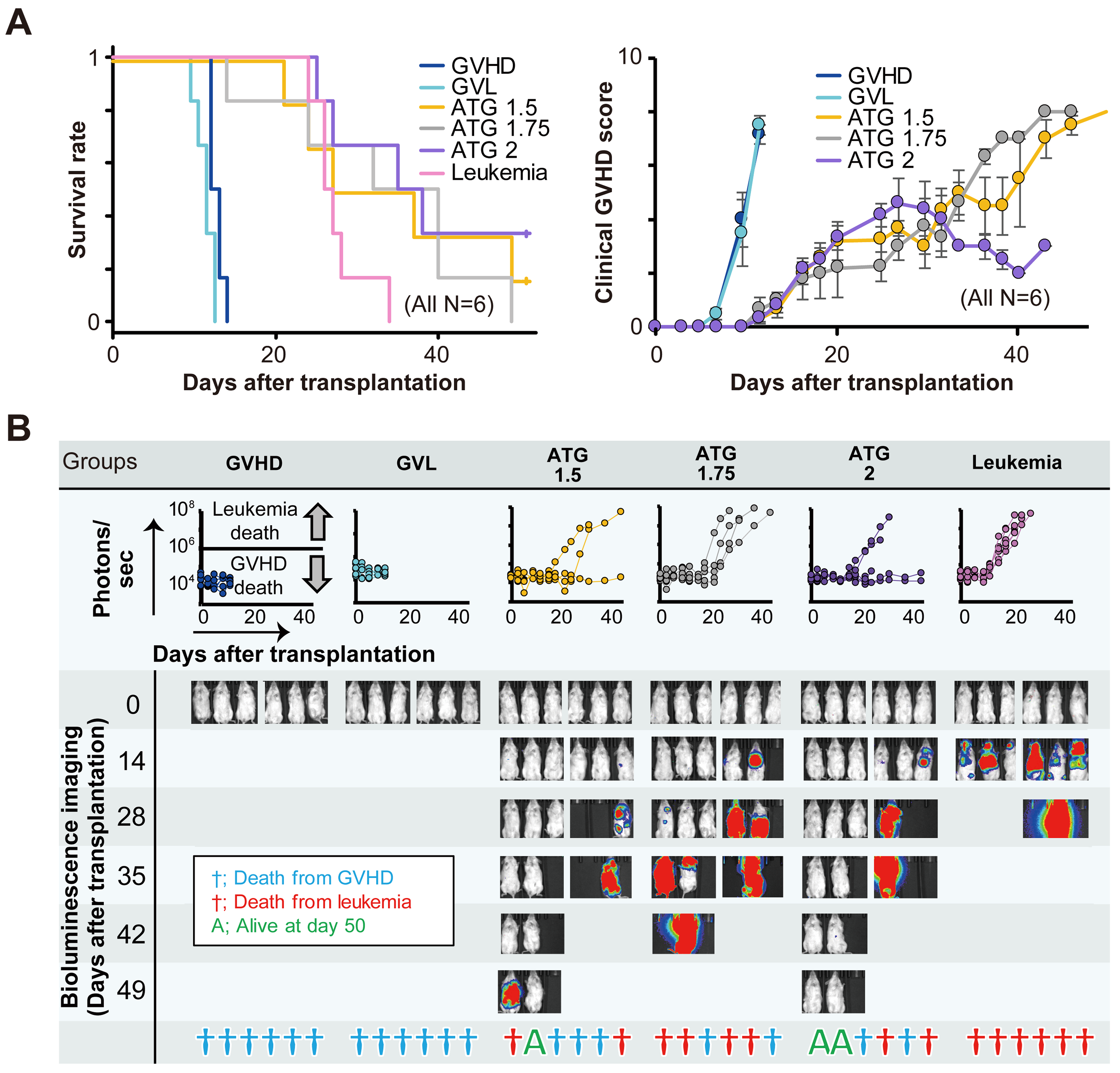

Supplement: S4 Fig — We compared ATG treatment at doses ranging from 1.25 to 2.5 mg/kg (1.5, 1.75, and 2.0 mg/kg). Each group consisted of six mice, and the mice were allocated to one of following six groups: GVHD, GVL with/without ATG treatment (1.5, 1.75, and 2.0 mg/kg), and leukemia. The mice were observed every day following transplantation for survival estimates and every other day to calculate their clinical GVHD scores (A). Bioluminescence imaging with photons (line graphs in the middle row) for each group and status of mice were observed for 50 days following transplantation (B). (TIF) [file pone.0245232.s004.tif]

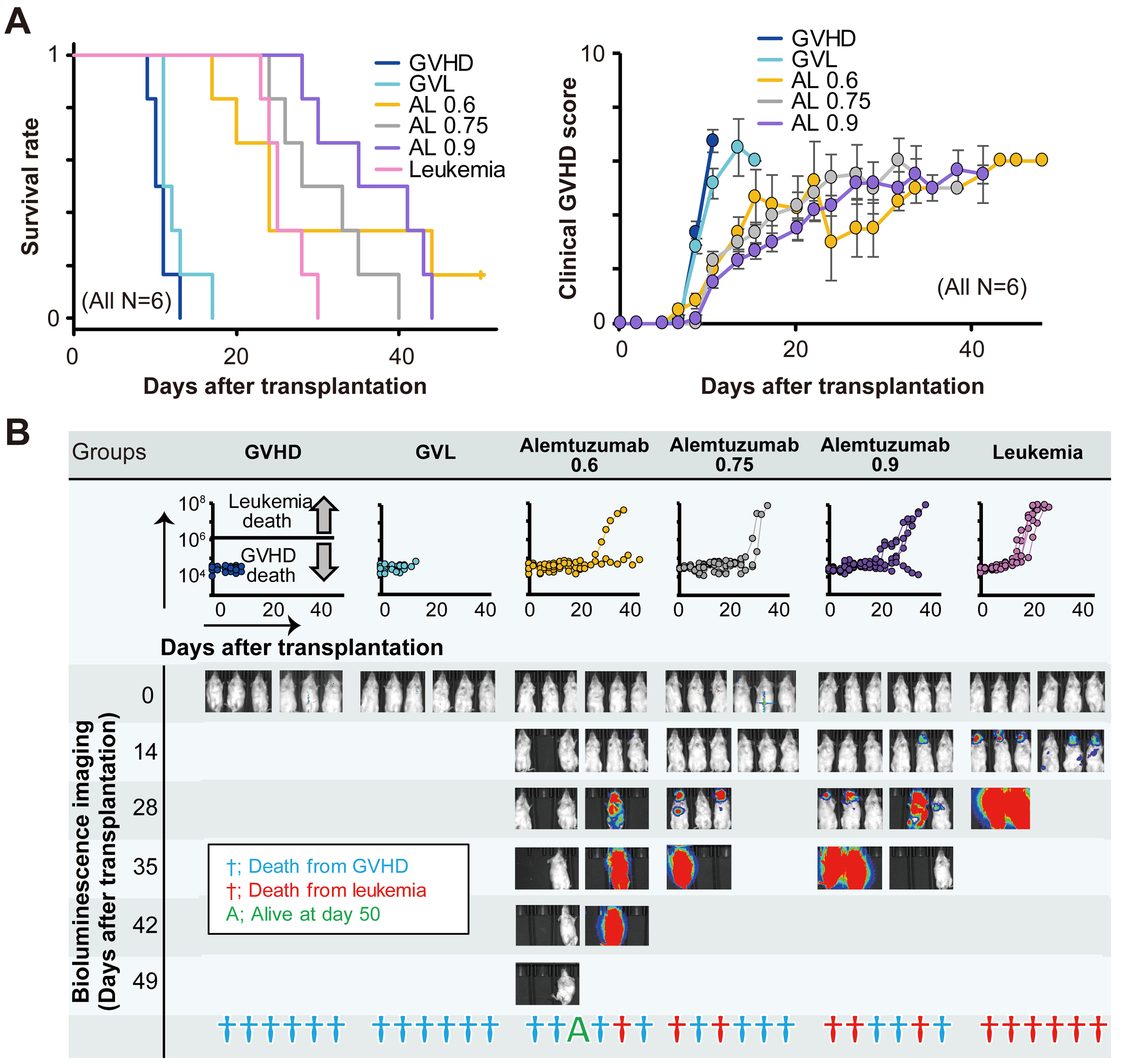

Supplement: S5 Fig — We compared alemtuzumab treatment at doses ranging from 0.5 to 1.0 mg/kg (0.6, 0.75, and 0.9 mg/kg). The mice were allocated to one of the following six groups: GVHD, GVL, alemtuzumab at 0.6, 0.75, and 0.9 mg/kg, and leukemia. Each group consisted of six mice, and they were observed every day following transplantation for survival estimates and every other day to calculate their clinical GVHD scores (A). Bioluminescence imaging with photons (line graphs in the middle row) for each group and status of mice were observed for 50 days following transplantation (B). (TIF) [file pone.0245232.s005.tif]
